# McKillip Veterinary College

**Published:** 1900-05

**Authors:** 


### McKILLIP VETERINARY COLLEGE.

THE fourth annual commencement of this college was held in the College Auditorium, 1639 Wabash Avenue, Chicago, Ill., on the evening of March 29, 1900, and the following programme presented :

Invocation, by Rev. Charles Brodie; baccalaureate address, by Dr. E. M. Reading; and class exercises, consisting of a salutatory address, by D. Clinton Burnett; class poem, by A. A. Adamson; class prophecy, by J. B. Hollenbeck; class history, by James Ragen; valedictory address, by J. P. O'Connor, followed the Secretary's report and presentation of prizes, and the conferring of degrees and the presentation of diplomas by President M. H. McKillip.

The following senior students received the degree of M.D.V.: S. E. Huntsberger, Burton City, Ohio; J. G. Hayes, Chicago, Ill.; Robert E. Cochrane, Milwaukee, Wis.; H. James Elliott, Brandon, Manitoba; Thomas E. Newell, Chicago, Ill.; M. J. O'Donnell, St. Clair, Pa.; James Ragen, Verona, Ill.; John E. Sommers, Buffalo, N. Y.; J. W. Rutledge, McGregor, Manitoba; A. A. Adamson, Newton, Iowa; D. Clinton Burnett, Sharon, Pa.; J. B. Hollenbeck, Salem, Ohio; S. P. Smith, Corasallis, Oregon; C. P. Liegerot, Ruxburg, Idaho; H. F. Pegan, Cochranton, Pa.; A. G. Hopkins, Madison, Wis.; James Nicholson, Pipestone, Minn.; S. G. Burkholder, Denver, Pa.; L. A. Merillat, Chicago, Ill.

The class of practitioners consisted of fourteen veterinarians, nine of whom passed the required examination, viz.: W. L. West, V.S, Bangor, Me.; Burton R. Rogers, D.V.M., Ames, Iowa; F. D. Markham, V.S., Port Leyden, N. Y.; S. P. Smith, D.V.M.; C. P. Liegerot, D.V.M.; H. F. Pegan, V.S.; D. C. Burnett, V.S.; J. B. Hollenbeck, V.S.; T. Lambreicht, V.S.

The auditorium was appropriately decorated by the junior class with flags and bunting; the attendance was large, and in the history of the college the fourth annual commencement will be recalled as a memorable event.

---

Dr. H. M. Hunter fills the rôle of veterinarian to Tulare County, California, and is now located at Visalia.

The ever-sanguine Pendry, of Brooklyn, while skilled in the treatment of the ailments of animals, is ever ready to prescribe for the ills of the human family, and while prescribing for others finds a quiet game the best cure for melancholia or a troubled mind, and a horseback ride a sovereign remedy for indigestion. He is wont to say "What a beautiful world this is, so full of fun, and yet how many people miss it." Pendry is never among the left overs.

